# *Sopele* music dataset

**DOI:** 10.1016/j.dib.2019.104840

**Published:** 2019-11-18

**Authors:** Arian Skoki, Sandi Ljubic, Jonatan Lerga, Ivan Štajduhar

**Affiliations:** University of Rijeka, Faculty of Engineering, Department of Computer Engineering, Vukovarska 58, 51000 Rijeka, Croatia

**Keywords:** *Sopele*, Woodwind instrument, Istrian peninsula, Automatic music transcription

## Abstract

Data presented in this article was created using a Croatian instrument called *sopela* – a traditional hand-made wooden aerophone of piercing sound, characteristic to the Istrian peninsula in western Croatia. The instrument is always played in pair (plural form: *sopele*), which consists of two voices: a *small sopela* and a *great sopela*. The data contains Waveform Audio File format (WAV) files, capturing every possible distinct tone of both *sopele*, as well as their polyphonic combinations. Additional data encompassed in the provided dataset are music scales and real music pieces, which contain specific traditional melodies. Every melody has a corresponding music sheet, presented in a Portable Document Format (PDF) file, which describes it in a human-readable manner. The specific Istrian scale music notation was applied while creating the music sheets. The data presented here was successfully utilised for developing, training and testing an automatic music transcription (AMT) solution, capable of converting *sopele* audio recordings into musical scores [1].

**Table:** Specifications Table

Subject area	*Computer science*
More specific subject area	*Machine learning for automatic music transcription*
Type of data	*Audio WAV files, PDF documents*
How data was acquired	*Embedded Mobile Phone Microphone, sampling rate of 44.*1 kHz
Data format	*Raw and analysed*
Experimental factors	*Combined tones were acquired by merging individual tones of the great sopela with the individual tones of the small sopela.*
Experimental features	*Recording of sopele was done by using an integrated mobile phone microphone. Recording position was varied during the experiment. All music pieces were performed by the same artist.*
Data source location	*Rijeka, Croatia, University of Rijeka, Faculty of Engineering, Department of Computer Engineering, Vukovarska 58, 51000 Rijeka, Croatia* *Latitude: 45.346837 | Longitude: 14.409897*
Data accessibility	*Data is with this article, along with the corresponding PDF music sheets.*
Related research article	*A. Skoki, S. Ljubic, J. Lerga, I. Štajduhar, Automatic music transcription for traditional woodwind instruments sopele, Pattern Recognition Letters, Volume 128, 2019, pp. 340–347,**https://doi.org/10.1016/j.patrec.2019.09.024*

**Table:** 

**Value of the Data** • This is the first publicly-available dataset of annotated music performed on sopela, an instrument formally recognised by UNESCO [2] as a part of intangible cultural heritage of humanity. • The sopela tones, both monophonic and polyphonic, were generated by the same artist, and were recorded from various positions during the experiment. The data include every possible distinct tone of both small sopela and the great sopela, as well as their polyphonic combinations. • Provided real-world music pieces, i.e. traditional songs, additionally include corresponding sheet representations. • The data can be used by researchers for developing, training and evaluating automatic music transcription (AMT) algorithms, stacked methods, and/or complete solutions. It is useful for comparing the quality and efficiency of different AMT algorithms for sopele-based sound, as well as for facilitating the development of new AMT solutions targeting woodwind instruments in general.

## 1 Data

The data consists of both raw and analysed audio recordings of music played on the instrument *sopela*. Both the *small sopela* and the *great sopela* were used for making these recordings. The data submitted alongside this article is structured as follows. Directory single_tones contains recordings of individual tones for both *sopele*. *Great sopela* tones are labelled {m0,m1,m2,m3,m4,m5}, whereas *small sopela* tones are labelled {v0,v1,v2,v3,v4,v5} (from a higher to a lower frequency pitch). Directory combined_tones contains all possible tone combinations between the *great sopela* and the *small sopela* individual tones. Directory real_pieces is divided into three categories: great_sopela, small_sopela, and combined_sopela. First two directories hold monophonic musical pieces for either the *great sopela* or the *small sopela*. The last directory contains stereo recordings containing both the *great sopela* and the *small sopela* melodies, playing simultaneously. All three directories contain multiple music files with a specific melody. Each melody has an accompanying music sheet in Portable Document Format (PDF). Music sheet is a human representation of a melody played. The sheet cannot be considered as ground truth; however, it is closest to it. Silence at the beginning and at the end of a recording is ignored, and is not showed in the sheet.

## 2 Experimental design, materials, and methods

**Fig. 1:**
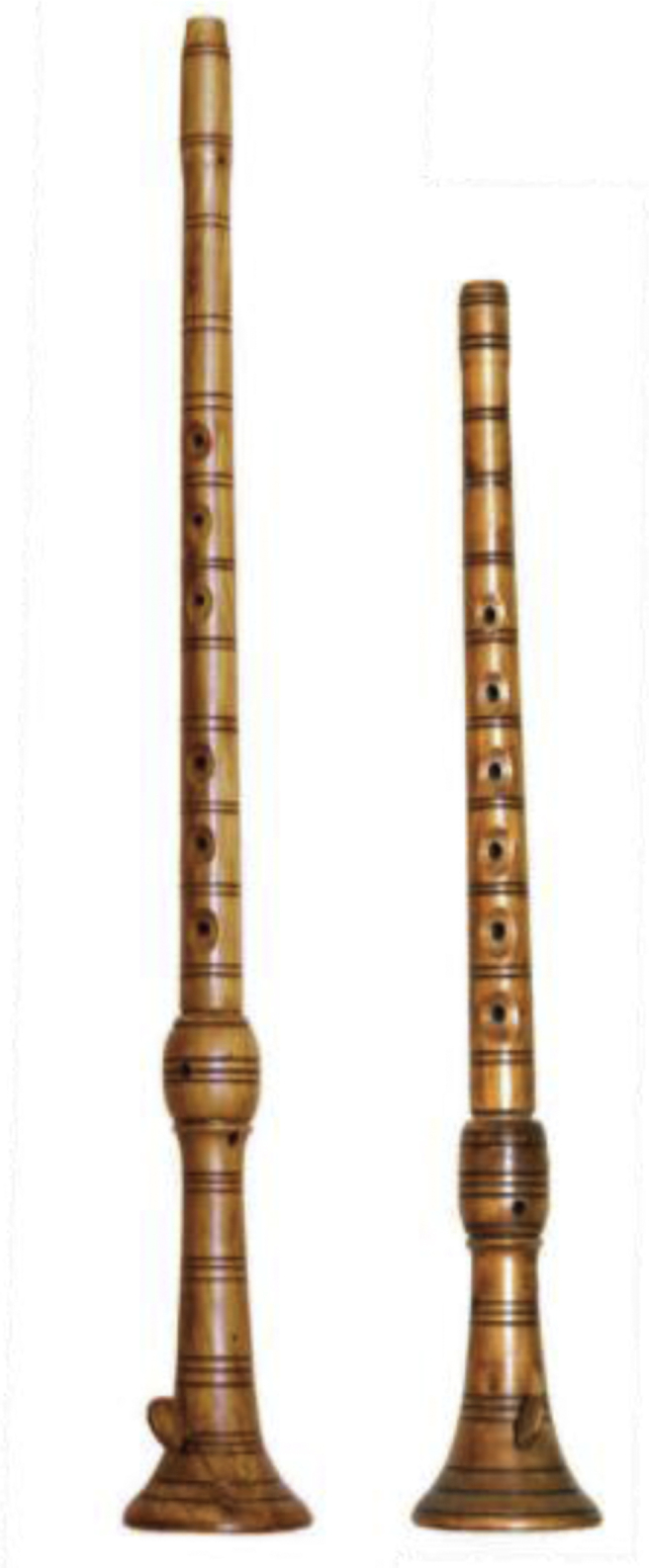
A pair of sopele [3]: great sopela (left), small sopela (right).

The experiment took place in a hallway, shaped like a letter L. Only one amateur *sopela*-playing musician was engaged in the process of data acquisition. The musician operated on the same pair of *sopele*. The pair of instruments used for producing audio is depicted in Fig. 1.

**Fig. 2:**
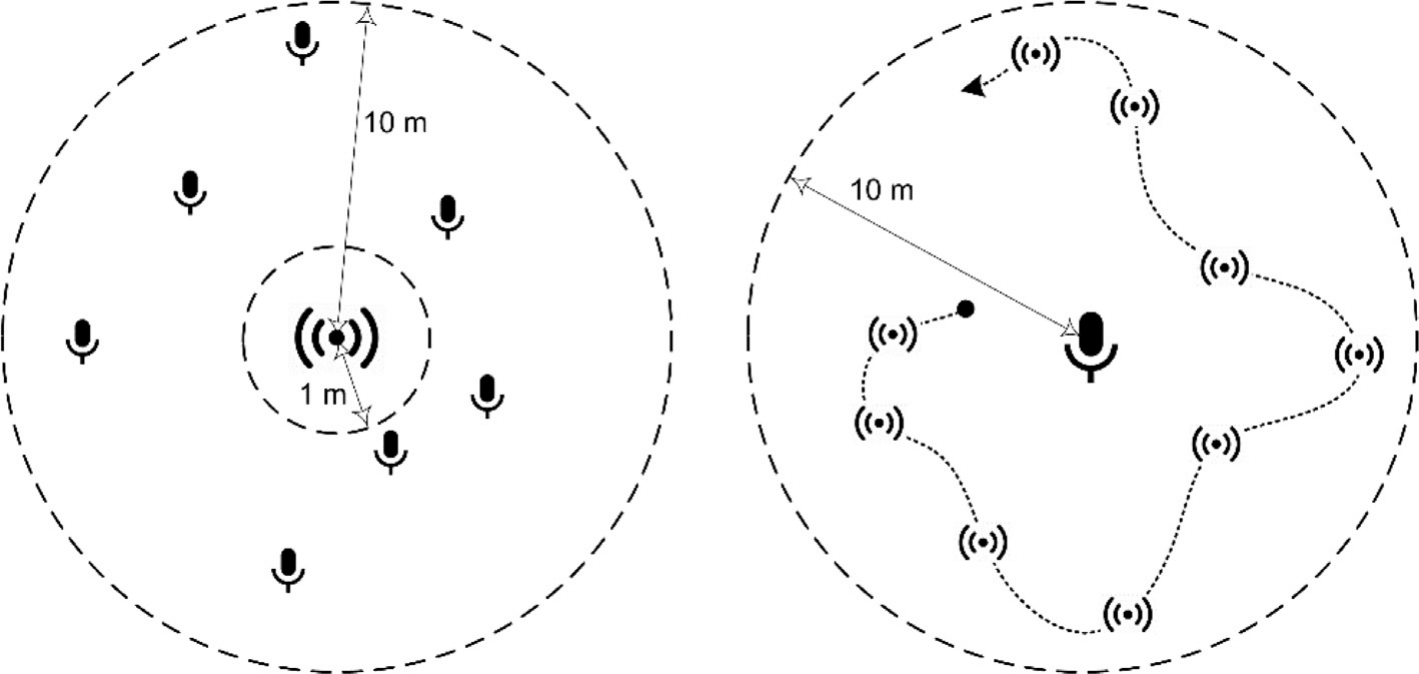
Audio recording settings: a musician at a fixed location with the microphone being positioned at different lengths (left); microphone at fixed location with the performer moving while playing the sopela (right).

Integrated Huawei P9 Lite microphone with sampling rate of 44.1 kHz was used as a recording device. Audio recordings representing single tones of either *great* or *small sopela* were acquired using several recording positions. The first set of recordings was gathered by statically positioning the microphone between 1 and 10 m from the audio source. In the second set, the microphone was placed at the source, however the performer was moving in range of 10 m while playing the instrument. These two settings (i.e. recording modalities) are illustrated in Fig. 2. In the end, every tone had 3-4 WAV files whose cumulative duration was between 21 and 38 seconds.

**Fig. 3:**
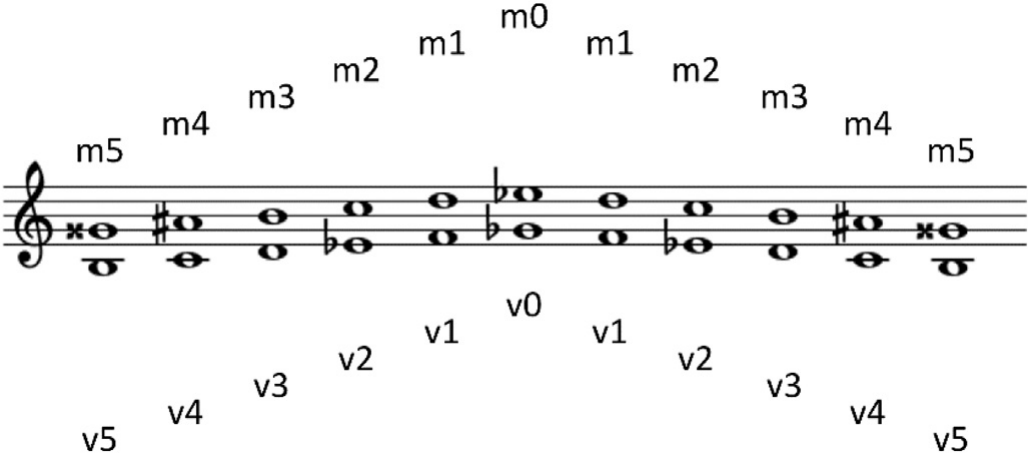
A depiction of the hexatonic Istrian scale. Two parallel note sequences, top and bottom, represent small and great sopela tones, respectively.

Each *sopela* can produce only six distinct tones, in line with the corresponding hexatonic Istrian scale (shown in Fig. 3). Hence, there were in total 12 distinct tones. Detailed information concerning the related recordings can be seen in Table 1.

**Table 1:** File characteristics of audio recordings and tonal distribution. For each tone, the black dot represents its assignment to either the great, or the small *sopela*.

Tone	Great *sopela*	Small *sopela*	Total duration [s]	Number of files
m0		●	26	4
m1		●	23	3
m2		●	28	3
m3		●	21	3
m4		●	36	4
m5		●	27	3
v0	●		28	3
v1	●		30	3
v2	●		38	3
v3	●		32	3
v4	●		30	3
v5	●		33	3

Combined tones (36 non-permutative combinations) of both *sopele* playing simultaneously were created by using existing single tones audio files. First, all audio files were merged into one, for each individual tone. After that, every tone, for both *sopele*, was recorded in one audio file. Audio files from the *great sopela* were merged with files from the *small sopela*, and saved into a new stereo file. If the recording duration of one file was longer than the other, surplus was discarded.

**Fig. 4:**
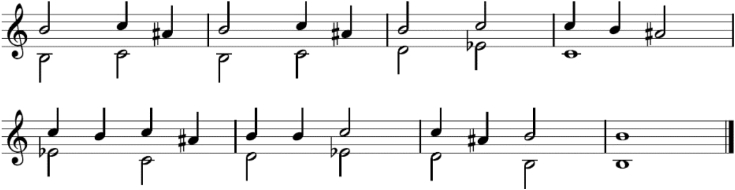
Music sheet provided alongside the audio recording, for the traditional song “Mare has been planting”. Both sopela sequences are presented on the same bar.

Real musical pieces, like the traditional wedding song called “*Sadila je Mare*” (roughly translated to “*Mare has been planting*”), containing more than one tone, were acquired by recording directly from the music source. Polyphonic recordings were created by merging *great sopela* and *small sopela* music files into one stereo file. For real musical pieces, accompanying musical scores are provided along with the audio data (example shown in Fig. 4).

The data presented here was successfully utilised for developing, training and testing an automatic music transcription (AMT) solution, capable of converting sopele audio recordings into musical scores [1].

## References

[1] Skoki A., Ljubic S., Lerga J., Štajduhar I. (2019). Automatic music transcription for traditional woodwind instruments *sopele*. Pattern Recognit. Lett..

[2] UNESCO Representative List of the Intangible Cultural Heritage of Humanity. https://ich.unesco.org/en/RL/two-part-singing-and-playing-in-the-istrian-scale-00231.

[3] Ethnographic Museum of Istria http://www.iti-museum.com/.

