# Supplementary material for: Sopele music dataset
Source: Data Brief. 2019 Nov 18;28:104840. doi: 10.1016/j.dib.2019.104840 (PMC6908999; doi:10.1016/j.dib.2019.104840)

Step 12

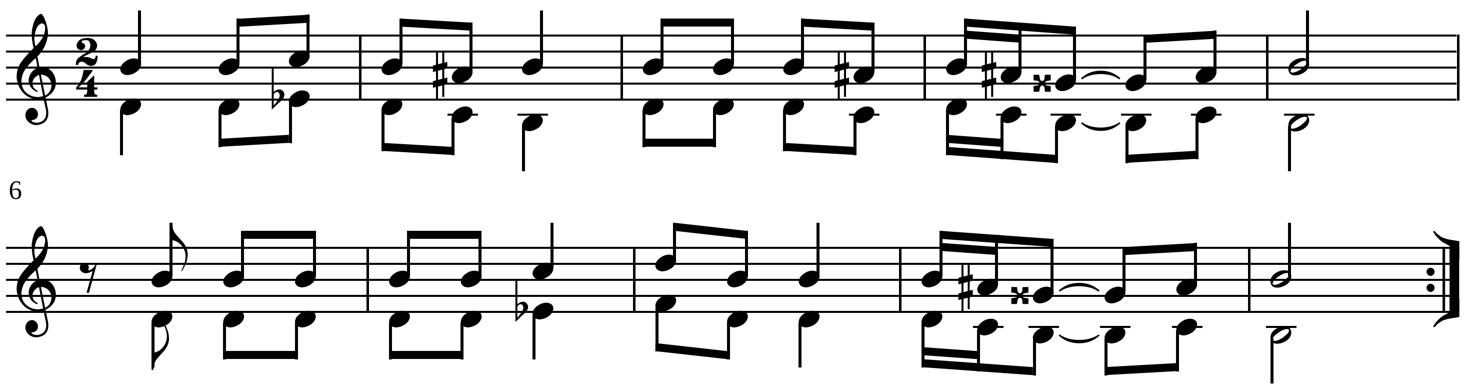

Supplement: Multimedia component 1 [file mmc1.zip › data/real_pieces/combined_sopela/Step_12/Step_12.pdf]
