# Supplementary figures and images for: Sopele music dataset
Source: Data Brief. 2019 Nov 18;28:104840. doi: 10.1016/j.dib.2019.104840 (PMC6908999; doi:10.1016/j.dib.2019.104840)

# Mare has been planting

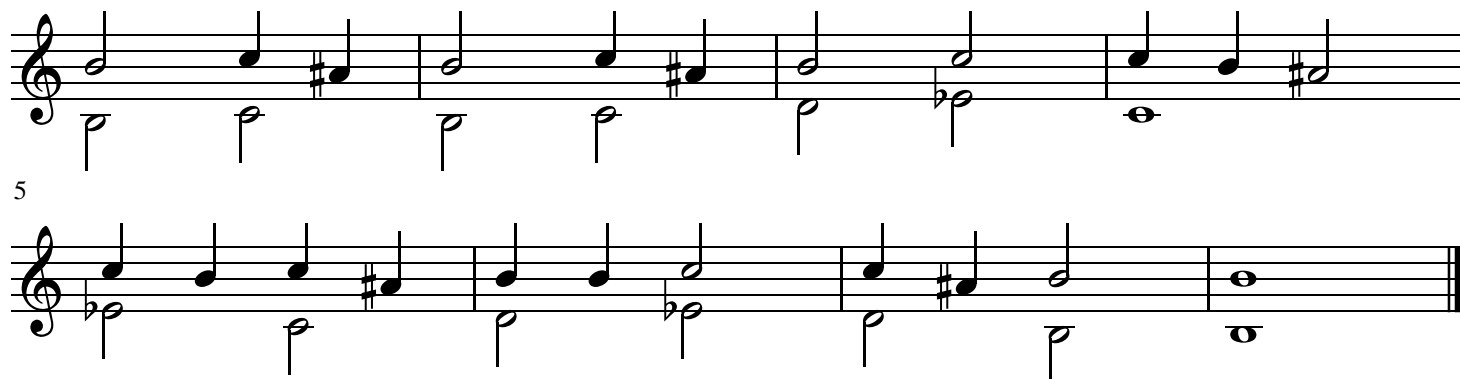

Supplement: Multimedia component 1 [file mmc1.zip › data/real_pieces/combined_sopela/Mare_has_been_planting/Mare_has_been_planting.pdf]

# Sopela full scale

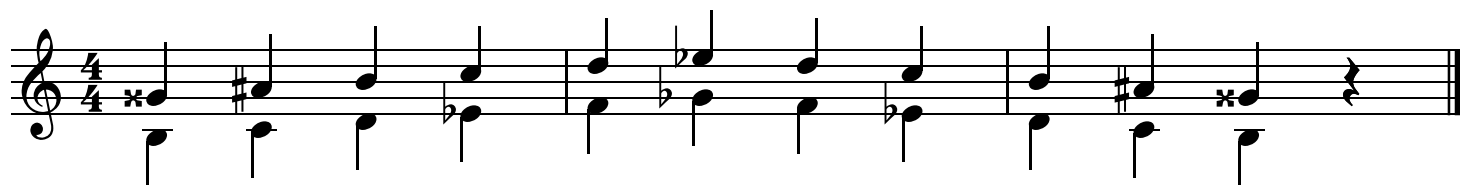

Supplement: Multimedia component 1 [file mmc1.zip › data/real_pieces/combined_sopela/Sopela_full_scale/Sopela_full_scale.pdf]

# Great sopela ascending scale

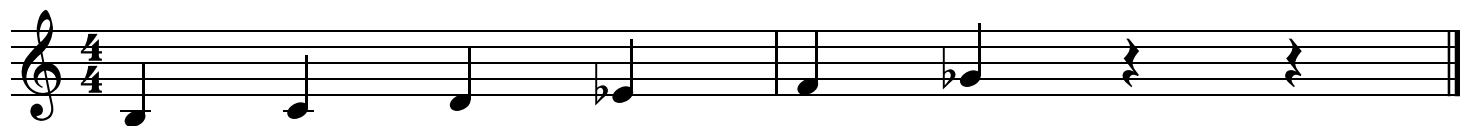

Supplement: Multimedia component 1 [file mmc1.zip › data/real_pieces/great_sopela/Great_sopela_ascending_scale/Great_sopela_ascending_scale.pdf]

# Great sopela descending scale

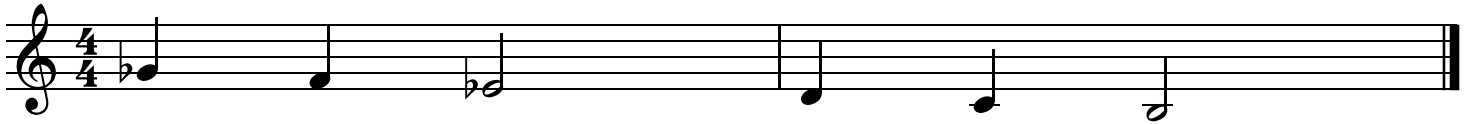

Supplement: Multimedia component 1 [file mmc1.zip › data/real_pieces/great_sopela/Great_sopela_descending_scale/Great_sopela_descending_scale.pdf]

# Great sopela full scale

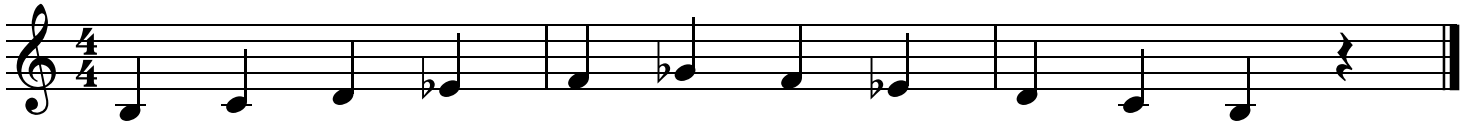

Supplement: Multimedia component 1 [file mmc1.zip › data/real_pieces/great_sopela/Great_sopela_full_scale/Great_sopela_full_scale.pdf]

# Great sopela full scale

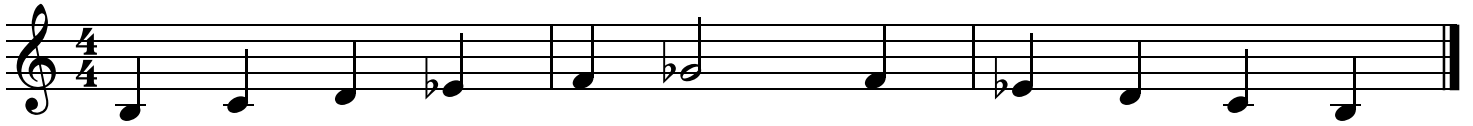

Supplement: Multimedia component 1 [file mmc1.zip › data/real_pieces/great_sopela/Great_sopela_full_scale_2/Great_sopela_full_scale_2.pdf]

# Great sopela quick ascending scale

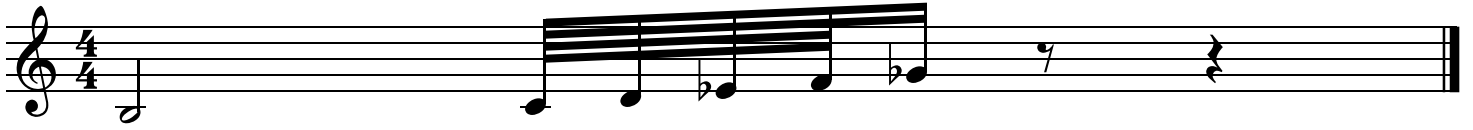

Supplement: Multimedia component 1 [file mmc1.zip › data/real_pieces/great_sopela/Great_sopela_quick_ascending_scale/Great_sopela_quick_ascending_scale.pdf]

# Mare has been planting - great sopela

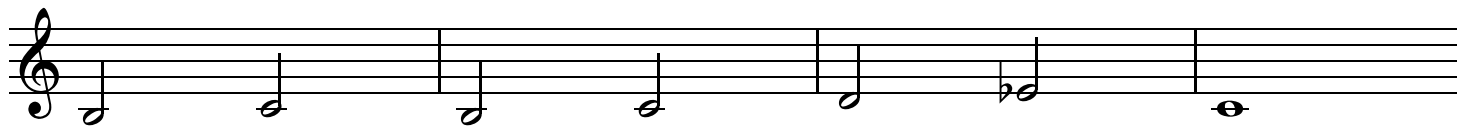

5

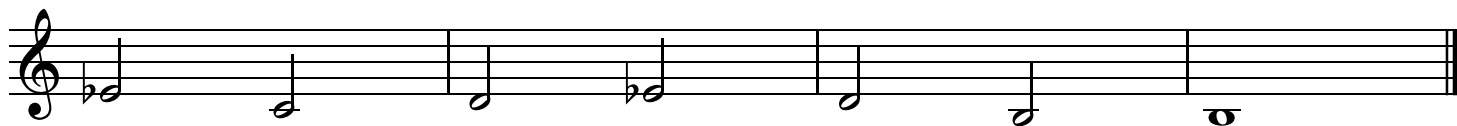

Supplement: Multimedia component 1 [file mmc1.zip › data/real_pieces/great_sopela/Mare_has_been_planting_great/Mare_has_been_planting_great.pdf]

Mare has been planting - small sopela

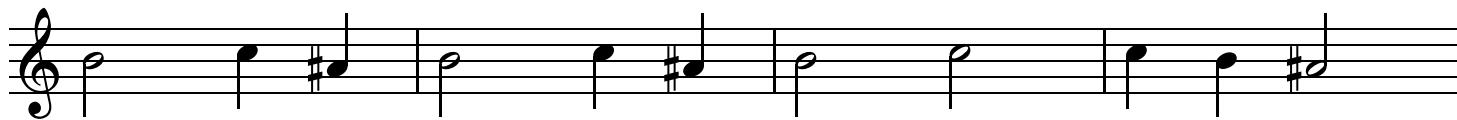

5

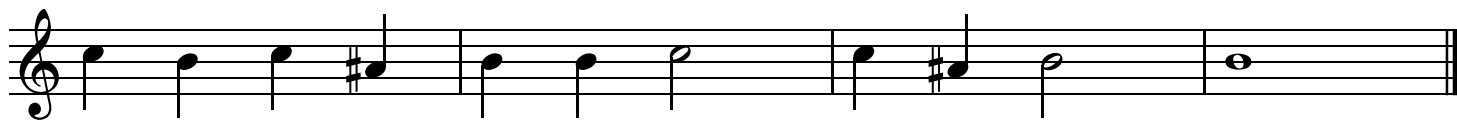

Supplement: Multimedia component 1 [file mmc1.zip › data/real_pieces/small_sopela/Mare_has_been_planting_small/Mare_has_been_planting .pdf]

# Small sopela ascending scale

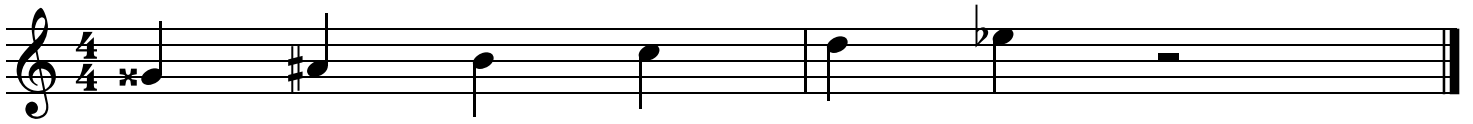

Supplement: Multimedia component 1 [file mmc1.zip › data/real_pieces/small_sopela/Small_sopela_ascending_scale/Small_sopela_ascending_scale.pdf]

# Small sopela ascending scale

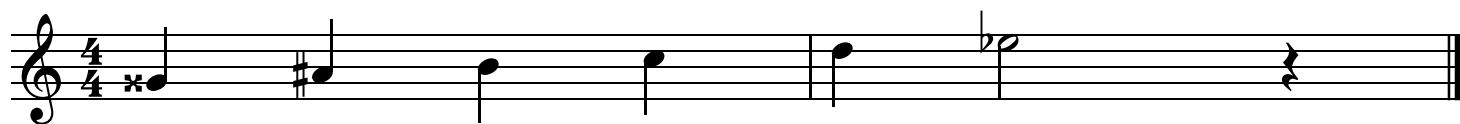

Supplement: Multimedia component 1 [file mmc1.zip › data/real_pieces/small_sopela/Small_sopela_ascending_scale_2/Small_sopela_ascending_scale_2.pdf]

# Small sopela descending scale

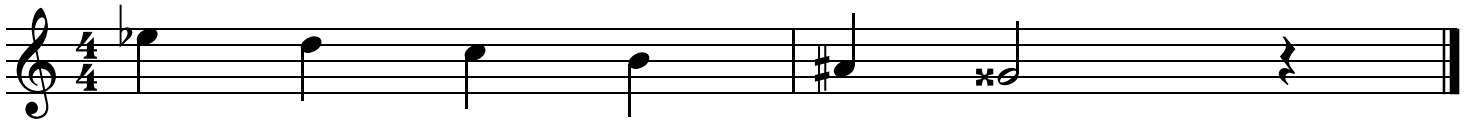

Supplement: Multimedia component 1 [file mmc1.zip › data/real_pieces/small_sopela/Small_sopela_descending_scale/Small_sopela_descending_scale.pdf]

# Small sopela full scale

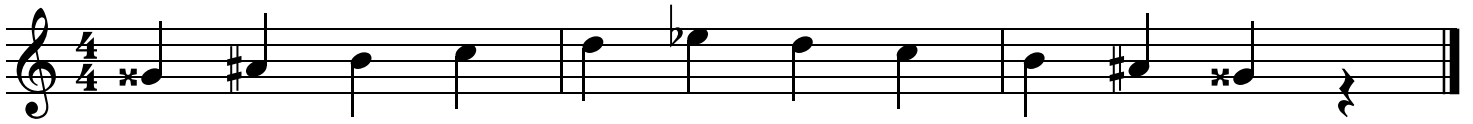

Supplement: Multimedia component 1 [file mmc1.zip › data/real_pieces/small_sopela/Small_sopela_full_scale/Small_sopela_full_scale.pdf]

## Small sopila quick ascending scale

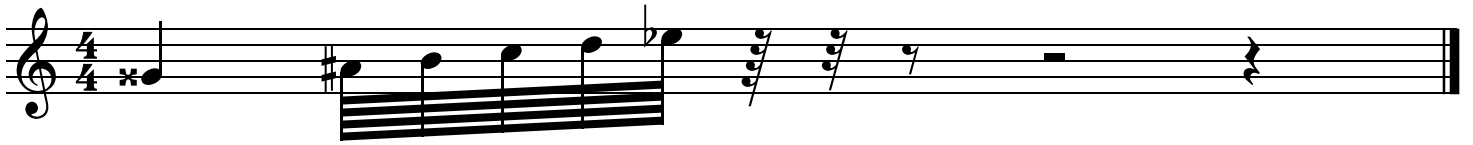

Supplement: Multimedia component 1 [file mmc1.zip › data/real_pieces/small_sopela/Small_sopila_quick_ascending_scale/Small_sopila_quick_ascending_scale.pdf]
